# Reimagining Resilience in Aging: Leveraging AI/ML, Big Data Analytics, and Systems Innovation

**DOI:** 10.1016/j.jagp.2025.05.007

**Published:** 2025-05-18

**Authors:** Jie Chen, Teagan K. Maguire, Rozalina G. McCoy, Stephen Thomas, Charles F. Reynolds

**Affiliations:** Department of Health Policy and Management (JC, TKM, RGM, ST), School of Public Health, University of Maryland, College Park, MD; School of Public Health (JC, TKM), The Hospital And Public Health InterdisciPlinarY Research (HAPPY) Lab, University of Maryland, College Park, MD; University of Maryland Center on Aging (JC, TKM, RGM); Division of Endocrinology (RGM), Diabetes and Nutrition, Department of Medicine, University of Maryland School of Medicine, Baltimore, MD; University of Maryland Institute for Health Computing (RGM), North Bethesda, MD; Center for Health Equity (ST), University of Maryland School of Public Health, College Park, MD; and the Geriatric Psychiatry (CFR), University of Pittsburgh School of Medicine, Emeritus Faculty, Pittsburgh, PA. Send correspondence and reprint requests to Jie Chen, Ph.D., Department of Health Policy and Management, Center on Aging, HAPPY Lab, School of Public Health, University of Maryland, College Park, MD 20742.

**Keywords:** Resilience, Depression, Aging health, Health disparities, Integration, Collaboration, AI/ML

## Abstract

As the aging population in the United States grows, the need for an integrated approach to support older adults has become increasingly urgent. The SUNSHINE framework, **S**eniors **U**niting **N**ationwide to **S**upport **H**ealth, **IN**tegrated Care, and **E**volution, offers a model for advancing resilience, defined as the capacity of individuals, families, systems, and communities to adapt and thrive in the face of adversity. SUNSHINE promotes this goal through the alignment of older and aging adults, families, healthcare systems, public health agencies, social services, and community resources. Using the Theory of Change modeling, SUNSHINE emphasizes whole-person health, interdisciplinary collaboration, and the strategic use of technology to address the evolving needs of aging populations. The framework promotes systems integration supported by research infrastructure and multi-sector collaboration to enhance the well-being of older adults and family caregivers. SUNSHINE places a strong emphasis on mental health, particularly depression, and highlights the importance of social connection and prevention in addressing health disparities and care gaps associated with aging. It conceptualizes resilience as both a desired outcome and a driver of transformation, guiding the redesign and evaluation of health and social systems. The framework also identifies opportunities to leverage artificial intelligence and machine learning (AI/ML) technologies, grounded in scientific evidence, to support personalized prevention, treatment, and care strategies. These technologies are critical for optimizing decision-making, improving care delivery, and enhancing system flexibility. Finally, SUNSHINE aspires to advance a future of aging that is healthy, resilient, and fair, guided by principles of equity, defined as fairness and impartiality in health opportunities and outcomes.

## INTRODUCTION

The United Nations Decade of Healthy Aging (2021−2030) calls for urgent, transformative solutions to meet the needs of a growing and increasingly diverse aging population. By 2030, individuals aged 65 and older are projected to constitute 20% of the U.S. population.^1,2^ Nearly 70%−80% of older adults live with multiple chronic conditions, including diabetes, cardiovascular disease, and dementia.^3,4^ These conditions place significant pressure on individuals, families, and healthcare systems, particularly within a fragmented U.S. healthcare delivery system characterized by limited accessibility, inconsistent quality, and high costs.^5–10^

Older adults with complex health needs experience disproportionately high rates of avoidable emergency department (ED) visits, hospitalizations, placement in long-term care, and fragmented care.^11–13^ These challenges are intensified by adverse social determinants of health (SDOH), such as poverty, inadequate housing, limited transportation, food insecurity, and insufficient access to health and social services.^5,11–18^ Systematic barriers to timely and quality care, especially among minority, low-income, and rural populations, further exacerbate disparities in physical and cognitive well-being, leading to higher disability and mortality rates.^5,12,13,19–23^ As a result, these intersecting challenges place a growing strain on an already overburdened and fragmented U.S. healthcare system.

Resilience, broadly defined as the capacity to adapt, maintain, or regain well-being in the face of adversity, offers a powerful lens through which to address many of these challenges.^24–26^ It reflects the dynamic interplay between physical and mental health, social support, and systemic resources that enable individuals and communities to thrive despite adversity.^15,27^ This concept encompasses both adaptation (e.g., adjusting to stressors) and augmentation (e.g., enhancing one’s ability to overcome them through targeted interventions).^26^ Resilience-building efforts have shown promise in improving whole-person health.^25,26,28–31^

To achieve meaningful improvements in aging outcomes, resilience must be supported not only at the individual level, but also across families, communities, and systems. Strengthening resilience requires interdisciplinary, evidence-based strategies that integrate clinical care, public health, research, social services, and community support.^32–34^ Indeed, multi-sector collaborations involving healthcare systems, housing authorities, transportation agencies, and public health departments have demonstrated effectiveness in advancing community-level well-being and sustainability.^35–40^

In this paper, we present the SUNSHINE framework as a strategic enactment of the Theory of Change (ToC) modeling processes to promote aging resilience through systems integration.^41,42^ We outline both the *what* (the vision and structure of integrated, resilient systems) and the *how* (the collaborative infrastructure, research processes, and innovations needed to achieve it). We describe the research infrastructure and collaborative approaches necessary to support SUNSHINE’s implementation and to strengthen resilience at the individual, organizational, and community levels. Our premise is that attention to these three levels is essential to optimizing the well-being of older adults in ways that are fair and impartial. In addition, we examine the evolution of artificial intelligence and machine learning (AI/ML) technologies in supporting older adult health and resilience. Furthermore, we address the challenges associated with the future application of AI/ML in this space, including the substantial investments required and the need to ensure that potential benefits are equitably realized. By linking technological innovation with system-level transformation, SUNSHINE aims to build a more adaptive, sustainable, and person-centered future for aging populations.

## THE SUNSHINE FRAMEWORK

**FIGURE 1.**
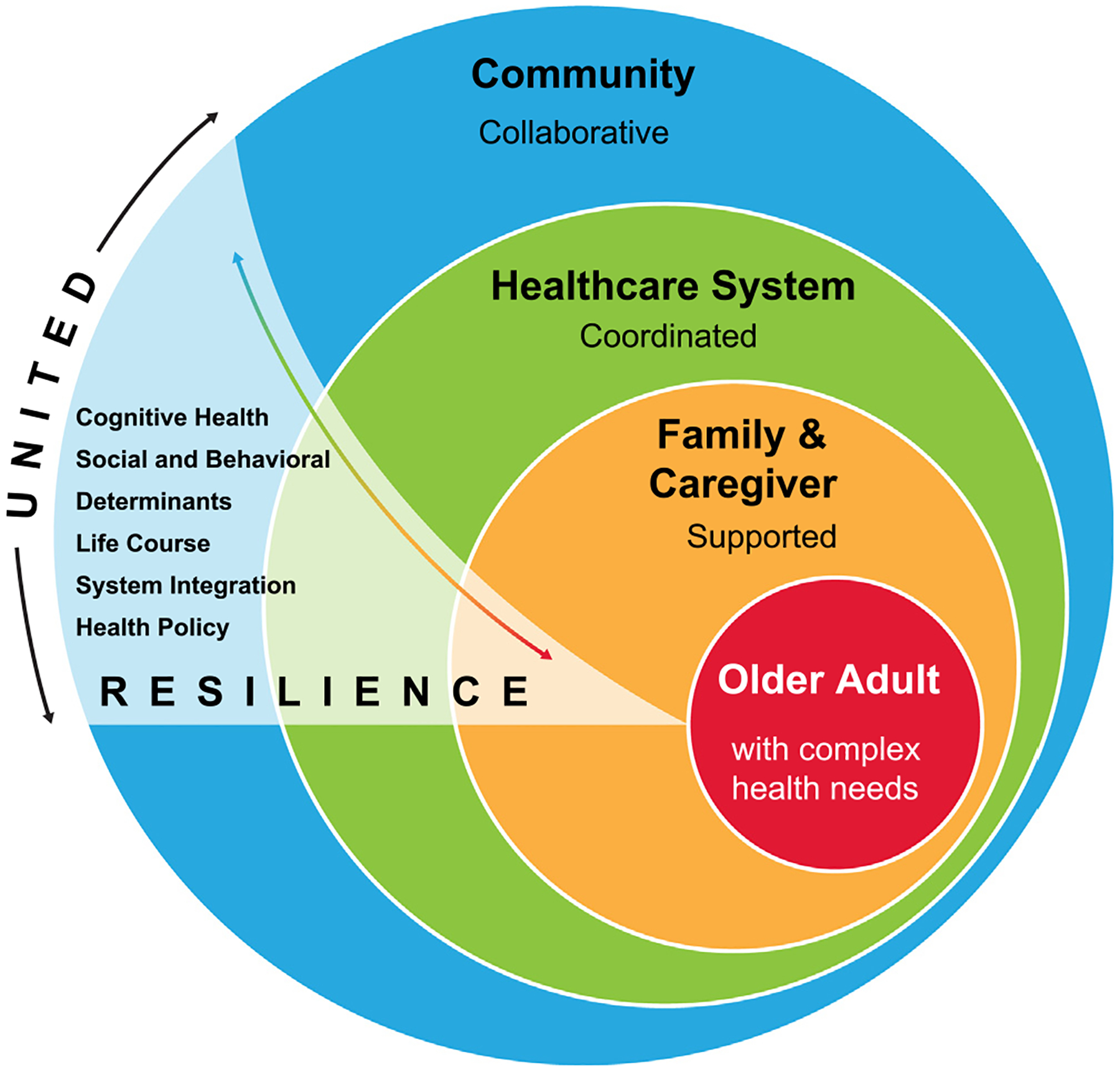
A resilient SUNSHINE framework that promotes whole-person health and whole-person care.

We propose the SUNSHINE framework, Seniors Uniting Nationwide to Support Health, Integrated Care, and Evolution, as a conceptual model to advance aging resilience through multi-level integration, equity, and innovation (Fig. 1). In this framework, resilience is defined as the capacity of individuals, families, systems, and communities to adapt and thrive in the face of adversity.^3,24,25,43,44^ SUNSHINE expands this concept to include system-wide resilience, emphasizing alignment across healthcare systems, public health infrastructure, social service agencies, and community-based organizations to meet the needs of older adults and their families. Its overarching goal is to improve well-being and maximize the societal value of older adult health by addressing modifiable risk factors and strengthening resilience at the individual, organizational, and community levels.^4,26,32,45–49^

SUNSHINE views resilience not only as an outcome but also as a driver of transformation.^50–52^ It supports a reimagining of aging populations as vital contributors to society.^26^ This vision is deeply informed by Martha Nussbaum’s capability model, which emphasizes that a person’s ability “to do and to be” depends not only on their internal capacities but also on the societal conditions and opportunities available to them.^53^ The model underscores the importance of creating environments where older adults can continue to lead purposeful lives, make meaningful choices, and share their accumulated wisdom and experience.^53^ Older adults bring a wealth of lived experience, intergenerational insight, and moral leadership that can enrich families, strengthen communities, and provide continuity during times of change. As Dworkin observed, they have helped shape the very fabric of society and offer unique perspectives, particularly during moments of social upheaval or moral uncertainty.^54^ Far from being peripheral, older adults are essential to sustaining cultural continuity, mentoring future generations, and guiding collective progress. Their wisdom, shaped by a lifetime of contributions, should be recognized, valued, and actively integrated into the core of civic and social life.

To put this vision into practice, the SUNSHINE framework is organized around four interconnected components: cognitive and behavioral health, social and behavioral determinants of health across the life course, system integration, and population-level policies and interventions. Each represents a key domain for building resilience.

1. Cognitive and Behavioral Health: SUNSHINE recognizes mind/brain health as a key lever for building resilience among older adults. This domain supports the development of models that promote cognitive, emotional, behavioral, and social well-being through interdisciplinary care, public health strategies, and community-integrated services. Key strategies include early screening, family-centered interventions, social prescribing, and a life-course approach to mental health.

Depression is a major threat to resilience, contributing to disability, diminished quality of life, and premature mortality.^29,55–57^ Yet it is also treatable and preventable through early detection and evidence-based intervention.^58–61^ A patient-focused, family-centered approach, grounded in the social determinants of well-being, is essential to supporting emotional and physical health.^62^ Social isolation further undermines recovery and resilience, particularly among critically ill older adults.^63^ In this context, social prescribing, referring individuals to community-based, nonclinical supports, offers a promising strategy to reduce depression, loneliness, and social disconnection.^64^ SUNSHINE supports the expansion of social prescribing models, especially when paired with trusted messengers such as peer support specialists and faith communities, which are known protective factors against depression and suicide, particularly in bereavement.^65^

2. Social and Behavioral Determinants of Health Across the Life Course: This domain examines how social, economic, and behavioral factors across the life course contribute to disparities in aging outcomes. These factors include cumulative disadvantage, adverse childhood experiences, and chronic stress exposure.^66,67^ SUNSHINE prioritizes identifying and addressing modifiable risk pathways, particularly those influencing mental health, resilience, and social connection. A life-course perspective is essential, as early-life adversity, including neglect and trauma, is strongly associated with poor mental and cognitive health outcomes in later life. SUNSHINE supports research and practices that address early-life adversity and promote well-being throughout life and into the later years of life.

3. Integration of Healthcare, Public Health, and Social Services: This domain examines how healthcare systems, public health agencies, and social services interact to support older adults in ways that are accessible and fair. SUNSHINE promotes integrated, whole-person models of care that bridge clinical and nonclinical supports, such as housing, transportation, food, and behavioral health, to improve care quality, coordination, and equity. System-level strategies that align service delivery with the broader social determinants of health are essential for sustainable and positive aging.

4. Impact of Population-Level Interventions and Policy: This domain evaluates how healthcare policies and system-wide innovations affect equity, resilience, and population well-being. It focuses on the role of evidence-based strategies, including AI/ML-enabled tools, value-based payment models, and cross-sector partnerships, in addressing geographic and cultural disparities in care delivery. This includes understanding how policy interventions shape brain and cognitive health outcomes by targeting modifiable risk factors, strengthening care coordination, and supporting integrated, person-centered care across clinical and community settings.

Emerging science continues to shape our understanding of brain and cognitive health, highlighting the impact of both clinical and social factors. The 2020 Lancet Commission on Dementia Prevention identified twelve modifiable risk factors for brain and overall health outcomes, including limited education, hypertension, smoking, obesity, depression, diabetes, and air pollution.^48,68,69^ Many of these are also contributors to accelerated brain aging, such as elevated blood pressure and allostatic load.^66,67^ Conversely, healthy lifestyle behaviors have been associated with delayed brain aging.^70^ In addition, treatment pathways, such as physical rehabilitation after stroke, can also improve mobility, enhance brain health, and slow cognitive decline.^71,72^

At the systems level, resilience is supported by care coordination models, strong public health infrastructure, and supportive built environments (e.g., housing and transportation).^73–76^ Cross-sector partnerships, particularly those involving public health agencies and healthcare systems, often facilitated by health information exchanges, are critical to integrating and sustaining care delivery.^77^ Nonclinical supports have also been shown to reduce delays in care and address unmet needs among older adults living with multimorbidity and cardiometabolic risk.^73,74^

Innovative financing mechanisms, such as value-based payment models (e.g., accountable care organizations), promote coordinated, person-centered care and alignment among providers, social service agencies, and community organizations.^75,76^ For example, ACO enrollment among Medicare beneficiaries with Alzheimer’s disease and related dementias has been linked to reduced spending, particularly for those living in disadvantaged communities.^78^ These findings suggest that value-based models can strengthen system resilience and fairness in managing chronic and complex conditions.

In summary, SUNSHINE calls for integration across healthcare delivery, public health systems, and community-based organizations to support older adults in a holistic, sustainable, and scalable approach. It reframes aging resilience as a shared societal responsibility, enabled through cross-sector collaboration, tailored interventions, applied technologies, and upstream prevention strategies. SUNSHINE advances the idea that resilience is not only an outcome of supportive systems but also a driver of transformation, shaping how we design, deliver, and evaluate care for aging populations. It also recognizes that the wisdom and contributions of older adults are essential assets, not only to the health of individuals and communities but to the resilience and progress of society as a whole.

## BRINGING SUNSHINE TO LIFE FROM A CLINICIAN’S PERSPECTIVE

Dr. Raya Kheirbek, Professor of Medicine and Chief of the Division of Gerontology, Geriatrics, and Palliative Medicine at the University of Maryland School of Medicine in Baltimore, Maryland, shared: *As a geriatrician in a community-based setting, I often see how fragmented systems fail to meet the complex needs of older adults managing multiple chronic conditions alongside social and emotional vulnerabilities. The SUNSHINE framework resonates deeply with these day-to-day realities —it reflects the kind of resilience-building infrastructure we try to piece together every day with limited, disconnected tools*.

I think of one patient, an 83-year-old woman living alone with diabetes, depression, and a history of food insecurity. When she began missing appointments, our EHR flagged her as high-risk. While we lacked a fully integrated system, we created a patchwork response guided by the very principles SUNSHINE promotes. A care manager connected her to a local nonprofit for food assistance. I reached out to a behavioral health colleague who scheduled a virtual visit. Most meaningfully, we activated a member of her faith community—contacted through a neighborhood network—who began visiting weekly. Although these efforts required manual coordination, the impact was substantial: her cognitive health improved, her mood stabilized, and emergency visits were avoided.

SUNSHINE envisions the kind of systemic alignment that would make these efforts scalable, sustainable, and equitable. It offers a roadmap to move from reactive care toward proactive, resilience-driven ecosystems—bridging what we currently improvise into what we could intentionally build together.

## BUILDING SUNSHINE RESEARCH INFRASTRUCTURE THROUGH THEORY OF CHANGE MODELING

Realizing the SUNSHINE vision requires a robust infrastructure that supports implementation, evaluation, and scalability. SUNSHINE emphasizes the iterative use, in collaboration with key stakeholders, of four foundational elements: big data, infrastructure, collaboration, and policy, that collectively advance a systems-oriented model of resilience in aging.

1. Integrated Data Systems: SUNSHINE supports the integration of administrative claims, survey data, electronic health records (EHRs), and geospatial datasets to enable a comprehensive, multidimensional view of aging, equity, mental health, and resilience. These linked data systems are essential for identifying population needs, measuring health and care gaps and disparities, and evaluating the impact of interventions across sectors.
2. Computational Infrastructure and Methods: Advanced analytical tools such as causal inference, geospatial analytics, econometrics, mixed methods, and AI/ML are key to generating timely, actionable insights. SUNSHINE encourages the development and application of rigorous computational methods to improve prediction, guide resource allocation, and evaluate interventions at multiple levels of care and community.
3. Interdisciplinary Collaboration and Innovation: SUNSHINE advances aging research through interdisciplinary partnerships that bring together public health experts, clinicians, engineers, economists, computer scientists, and social scientists. This collaborative model promotes the development of innovative, scalable solutions, such as AI-driven care optimization and systems modeling, to enhance care delivery, improve efficiency, and support system-level transformation. SUNSHINE promotes collaboration across academic, clinical, public health, and community sectors to address aging as a complex, multidimensional challenge and to support personalized, whole-person care that reflects the unique medical, social, and emotional needs of older adults and their care partners.
4. Community and Policy Engagement: SUNSHINE prioritizes grounding research in lived experience and ensuring that findings inform policy and practice. Meaningful stakeholder engagement, particularly among older adults, caregivers, community leaders, and policymakers, ensures that solutions are culturally responsive, locally relevant, and scalable. This pillar emphasizes the importance of codesign, shared ownership, and alignment with real-world needs and capacities.

These four pillars, integrated data systems, advanced computational methods, interdisciplinary collaboration, and community and policy engagements, support SUNSHINE’s vision of a comprehensive, system-oriented model for promoting resilience in aging. As one of the most promising frontiers in this work, AI/ML presents transformative opportunities to enhance aging resilience at every level, from individual care to system-wide innovation.

## EVOLUTION: MAXIMIZING TECHNOLOGICAL INNOVATION TO IMPROVE RESILIENCE

AI/ML tools are reshaping how we understand and support healthy aging. AI/ML tools that support early disease detection, personalize interventions, and real-time monitoring with rapid care team engagement can enhance individual resilience by help older adults adapt and recover. Meanwhile, AI/ML-powered tools that provide social companionship and promote autonomy can increase resilience by enabling older adults to maintain well-being and thrive despite changes in mental or physical health. AI/ML tools are increasingly used at the organizational level to reduce physician burnout, streamline administrative tasks, and support workforce and capacity planning.^75^ At the system level, SUNSHINE can benefit from applying AI/ML to identify sustainable, scalable payment and organizational models that better align incentives with health and well-being. AI/ML may also be useful in designing healthy aging communities and building stronger tools that detect risk at the individual and community levels, helping to direct the distribution of resources more effectively.

### Detection, Prediction and Personalized Health Interventions

AI/ML’s predictive and diagnostic capacities, particularly when paired with personalized health interventions, have the potential to increase resilience by enabling proactive, individualized care, early intervention, and prevention.^76^ While additional research is needed, substantial progress is already underway, supported by a growing body of robust studies. For example, in the context of neurodegenerative diseases such as Alzheimer’s disease (AD), early diagnosis is critical for slowing symptom progression and preserving functional independence.^79^ A broad range of AI/ML approaches is currently being tested for early identification of AD, including natural language processing to analyze patient speech, identification of peripheral biomarkers of risk, and convolutional neural networks identifying pathological changes in brain images in order to classify AD, MCI, and normal aging.^52,76,80–82^

AI/ML also assists clinical teams in optimizing medication dosages and combinations to reduce the risk of adverse drug interactions—a particularly urgent concern for older adults requiring polypharmacy.^76,83–86^ In both oncology and psychiatry, AI/ML tools that analyze EHR and molecular data are being used to tailor therapies to individual patient profiles.^87,88^ Predictive modeling is also being used to stratify risk and identify patients at greatest risk for medication-related harm.^89^

Beyond chronic disease management, AI/ML tools are increasingly being used to the predict risk of acute and sudden health events.^76^ For instance, in outpatient settings, AI/ML tools can predict adverse events such as heart attacks, heart failure, and diabetes-related complications. In hospital settings, these tools have also contributed to faster and more accurate detection of sepsis.^76,90–94^

### Wearable Technologies, Remote Patient Monitoring, and Telemedicine

Remote patient monitoring technologies (RPM) and wearable technologies have tremendous potential to augment traditional care and improve whole-person health, theraby enhancing resilience. Although RPM and wearable technologies are used narrowly within the healthcare system, a systematic review by Serrano et al. found consensus among healthcare practitioners that RPM strategies will become increasingly relevant.^95^ Devices such as smartwatches, fitness trackers, and sensor-equipped clothing and accessories like remote blood pressure monitoring systems can monitor vital signs, detect falls, assess sleep quality, and track physical activity.^76^ The data collected through these devices can be analyzed using AI/ML to improve preventive care. For example, AI algorithms can personalize health interventions, including fitness or nutrition plans, and can trigger rapid responses from care teams or emergency services, particularly in cases of fall detection or concerning changes in vital signs.^76^ RPM technologies also empower patients with real-time insights into their health and enable providers to monitor individuals with chronic or temporary conditions more effectively.^95^

Successful RPM deployment requires data collection, data transmission and storage, data analysis, and information presentation.^96^ Telemedicine, whether synchronous or asynchronous video, audio, or written communication, is often a component of RPM program information presentation.^97^ It is also an important component of healthcare delivery independent of RPM programming, particularly for older adults, who use telemedicine more than any other age group and report high satisfaction and positive experiences.^98,99^ Telemedicine has proven especially valuable in rural and underserved communities, as it can increase access to care, and has been associated with positive outcomes and satisfaction.^100–102^ Policy-makers continue to debate whether and how to permanently extend COVID-19 era regulatory flexibilities that made telehealth more feasible; incremental extensions and re-introduction of the Creating Opportunities Now for Necessary and Effective Care Technologies (CONNECT) Act in 2025 indicate telehealth will continue to be an important mode of care delivery.^103^

### Robotics, Health Agents and Smartphone Applications

AI/ML tools can also serve as social companions, provide engagement geared at improving mental health, and monitor and support older adults living independently. In fact, the top 10 generative AI use cases in 2025 published by Harvard Business School lists therapy/companionship at the top.^104^ One example is ElliQ, an AI-powered social robot developed to engage older adults through conversation and activity prompts. Early evidence suggests that ElliQ may reduce loneliness: 80% of test users reported feeling less lonely with the robot, and 74% said their quality of life had improved.^105^ Although ElliQ is not classified as a medical device, it illustrates the potential for consumer-facing technologies to positively influence health and well-being.^105^ Another example is Astro, a home robot developed by Amazon. Marketed as a home “companion,” Astro features fall detection, medication reminders, and remote caregiver access—merging emotional support with clinical utility.^106^ However, such social robots remain rare and costly; for example, Astro is currently available by invitation only at a price of $1,599.99.^107^

More widely adopted technologies, such as smartphones, also show promise. In a recent estimate, 61% of older adults in the U.S. own a smartphone.^106^ Research has demonstrated that smartphone apps can help older adults manage mental health, loneliness, and symptoms of dementia.^108,109^ For example, a randomized controlled trial by Scullin et al. found that smartphone tools effectively enhanced daily prospective memory and instrumental activities of daily living (IADLs) in older adults with dementia.^108^

AI/ML integration into smart-home technologies has the potential to enhance resilience for community-dwelling older adults. These systems are evolving from basic assistive devices to intelligent platforms featuring ambient intelligence, Internet of Things (IoT), and ambient assisted living (AAL) technologies. These innovations target various domains, including health monitoring, medication management, fall detection, support for IADLs, safety, entertainment, and energy savings.^9,110,111^ Smart-home technologies could have a tremendous impact on the continuity and quality of older adult health care through monitoring vital signs and supporting medication management, IADLs, fall detection, and quality of life.^112^

While preliminary findings are promising, further research is needed to evaluate the long-term effectiveness, ethical implications, and real-world usability of these technologies.^105^ A qualitative study by Wong et al. of older adult perceptions of AI/ML health technologies found that older adults saw value in AI/ML but continued to value human connection, and underscored the importance of AI as a complement, rather than a replacement, to human interaction.^113^

### The Future of AI/ML in Supporting Older Adult Health and Resilience

AI/ML technologies hold great promises for enhancing resilience among older adults, as well as the organizations and systems that serve them, but their full potential depends on addressing several critical challenges. First, misalignment in AI/ML development priorities across stakeholders hampers progress in AI/ML development, implementation, and impact.^114,115^ Convening stakeholders across healthcare, public health, policy, technology, community organizations, and older adults and care partners is essential to identifying shared priorities for leveraging AI/ML to influence population health and drive system- and community-level transformation. For example, AI/ML could be harnessed to inform the design of age-friendly communities, particularly in underserved areas, by helping identify local risk factors, predict service needs, and optimize resource allocation.

Second, longstanding inequities in access to healthcare technology raise concerns that AI/ML tools may worsen existing disparities. Studies have already documented disparities in AI/ML tool implementation, particularly in rural and under-resourced hospital settings.^77,78^ Meanwhile, a proliferation of stand-alone direct-to-consumer AI/ML-supported diagnostics and care optimization startups threatens to widen health disparities, as many of these businesses offer private diagnostics and memberships at unaffordable costs, with lengthy waitlists.^116,117^ Pay-for-play patient access to AI/ML tools is also occurring in routine preventive care as certain imaging labs ask mammogram patients whether they wish to pay out-of-pocket for AI/ML detection tools to evaluate their breast images for early indicators of cancer not discernable to radiologists.^118^ To avoid deepening health inequities, regulatory frameworks must prioritize equitable access to high-quality, AI-enabled care, especially for lower-income and historically underserved populations.

Third, there is an urgent need for more research on the clinical effectiveness and return on investment of AI/ML tools under both fee-for-service and alternative payment models.^115^ Expanding AI-enabled care through value-based payment models may help close gaps in access to technology innovations and quality healthcare. New Medicare-focused primary care entrants that operate under alternative payment models and blend traditional and at-home care, employ remote patient monitoring, and offer social services (e.g., transportation, social programming) align financial incentives with whole-person health and well-being.^119^ These models have the potential to advance resilience through scalable, sustainable system-level innovations.

Realizing the full and equitable benefit of AI/ML in gerontological science and clinical geriatrics will require interdisciplinary research, thoughtful regulation, multi-stakeholder collaboration, and user-centered design that promotes the needs and perspectives of older adults. When developed and deployed ethically, these technological innovations have the potential to support resilience across the aging continuum at all domains of the SUNSHINE resilience construct.

## CONCLUSION

Technological and policy innovations have made the SUNSHINE framework feasible. Digital tools, such as health information exchanges, EHRs, and big data analytics, facilitate the merging of clinical and public health data, enabling precision public health approaches tailored to community and individual needs.^120–122^ On the policy front, initiatives such as Medicaid 1115 waivers allow states to expand non-medical services, including housing, transportation, and food assistance, for older adults.^123,124^ The CMS GUIDE Model, launched in 2023, offers critical support for family caregivers and enhances care for individuals with serious illnesses, including dementia.^125–128^ The Inflation Reduction Act improves affordability by lowering prescription drug costs for Medicare beneficiaries, while the 21st Century Cures Act incentivizes the adoption of AI/ML tools to accelerate data-driven innovation in healthcare.^129,130^

In response to these converging opportunities and enduring challenges, the SUNSHINE framework offers a comprehensive, systems-oriented approach to advancing resilience in aging. By positioning resilience as both an outcome and a catalyst for transformation, SUNSHINE provides a scalable, real-world model for integrating healthcare, public health, social services, and community-based supports. This framework paper introduces SUNSHINE as a strategic model and presents a narrative review of the emerging role of AI/ML in supporting aging resilience at multiple levels of care and system design.

SUNSHINE places a strong emphasis on mental health conditions such as depression and highlights the critical role of social connection and prevention in addressing the root causes of aging-related disparities. It positions resilience not only as a desired outcome but also as a driver of transformation, a foundation for redesigning systems and measuring their impact. SUNSHINE promotes environments that promote well-being, independence, and longevity for all. The framework calls for the strategic use of technology, particularly AI/ML, grounded in rigorous scientific research to enhance aging health. In doing so, SUNSHINE lays the foundation for a more resilient, equitable, and connected future for older adults and their care partners. It provides a forward-looking roadmap for advancing aging science that is inclusive, actionable, and responsive to both current and emerging challenges.

## Data Availability

The data has not been previously presented orally or by poster at scientific meetings.

## Highlights

- **What is the primary question addressed by this study?**
  The study proposes The SUNSHINE framework—**S**eniors **U**niting **N**ationwide to **S**upport **H**ealth, **IN**tegrated Care, and **E**volution—for advancing resilience, defined as the capacity of individuals, families, systems, and communities to adapt and thrive in the face of adversity.
- **What is the main finding of this study?**
  The framework identifies four integral research domains and opportunities to leverage artificial intelligence and machine learning technologies. SUNSHINE envisions a resilient and evolving future of aging, where AI/ML innovations are integrated into care models that thoughtfully combine technology, community-based support, and value-based care to enhance well-being across the life course.
- **What is the meaning of the finding?**
  SUNSHINE conceptualizes resilience as both a desired outcome and a driver of transformation, guiding the redesign and evaluation of health and social systems, and emphasizes whole-person health, interdisciplinary collaboration, and the strategic use of technology to address the evolving needs of aging populations.
